# Internalizing Symptom Profiles Among Youth in Foster Care: A Comparison Study

**DOI:** 10.3389/fpsyt.2021.711626

**Published:** 2021-08-16

**Authors:** Yasmin Moussavi, Kyrre Breivik, Gro Janne Wergeland, Bente Storm Mowatt Haugland, Marit Larsen, Stine Lehmann

**Affiliations:** ^1^Regional Centre for Child and Youth Mental Health and Child Welfare—West, NORCE Norwegian Research Centre, Bergen, Norway; ^2^Department of Health Promotion and Development, Faculty of Psychology, University of Bergen, Bergen, Norway; ^3^Department of Child and Adolescent Psychiatry, Division of Psychiatry, Haukeland University Hospital, Bergen, Norway; ^4^Department of Clinical Medicine, Faculty of Medicine, University of Bergen, Bergen, Norway; ^5^Department of Clinical Psychology, Faculty of Psychology, University of Bergen, Bergen, Norway

**Keywords:** anxiety, depression, internalizing, youth in foster care, adolescents, latent profile analyses, assessment

## Abstract

**Background:** A high prevalence of anxiety and depression is found among youth in foster care. There is limited knowledge on the anxiety and depression symptom profiles of youth in foster care. We examined latent profiles of anxiety and depression symptoms across three unique youth samples and whether youth in foster care were more or less likely to belong to specific symptom profiles than their peers recruited from clinical or general youth populations. We also investigated if these profiles were predicted by sex and age.

**Methods:** Self-reported anxiety and depression symptoms were assessed by Spence Children's Anxiety Scale and Short Mood and Feelings Questionnaire. Data were pooled from three youth samples (*N* = 2,005; mean age = 13.9 years, range = 11–18 years) comprising youth in foster care (*n* = 245), a clinical youth sample (*n* = 107), and a general population youth sample (*n* = 1,653). Symptom profiles were identified using latent profile analyses. Multinominal logistic regression was used to predict the latent profile membership.

**Results:** Three profiles that differed both in symptom level and shape were identified and labeled as low, medium, and high symptom profile. Compared to the general population youth sample, youth in foster care had a higher likelihood of belonging to the high symptom profile, but not the medium symptom profile. Youth from the clinical sample had an increased risk of belonging to the medium and high symptom profiles compared to the youth in foster care and general population youth samples. Across samples, girls yielded a higher likelihood of having a medium or high symptom profile. Increasing age was associated with a higher likelihood of being in the high symptom profile.

**Conclusion:** Compared to their counterparts in the general population, youth in foster care are at risk of belonging to a class of youth with high symptom levels across subtypes of internalizing symptoms, indicating the importance of systematic and broad assessment of internalizing symptoms among these youth. Knowledge on the symptom profiles of anxiety subtypes and depression increases our understanding of the treatment needs of youth in foster care.

## Introduction

Anxiety and depression are among the most prevalent mental disorders in youth (1). The estimated worldwide point prevalence of any anxiety and depressive disorder in youth is 6.5 and 2.6%, respectively (2), with a higher prevalence among girls than boys (3, 4). These disorders may impede development and well-being (1, 5, 6) and are associated with numerous negative mental health outcomes (7, 8).

Robust empirical data exist on the prevalence of anxiety and depression (hereafter internalizing disorders) in both population-based youth samples (2, 9) and clinical populations (4, 10). While research on the prevalence of internalizing disorders among youth in foster care is scarce, a meta-analysis estimated a pooled prevalence of 11% for any depressive and 18% for any anxiety disorder among youth in the child welfare system (11). Importantly, the comorbidity of mental disorders is high among youth in foster and residential care (12, 13). Comorbidity is found to be a negative predictor of treatment outcomes for internalizing disorders in clinically referred youth (10). However, there is a dearth of research on the profiles of depressive symptoms and anxiety symptom subtypes among youth in general and especially among youth in foster care. This leaves us unaware of whether the likelihood of experiencing various symptom profiles is similar or dissimilar across different groups of youths. Knowledge on load and distribution of symptoms across a wide range of internalizing problems among youth in foster care may have clinical implications for the conceptualization, assessment, and treatment outcomes of internalizing problems among at-risk youth, such as those in foster care.

Studies investigating the comorbidity of anxiety and depression are usually variable-centered, focusing on the associations of the variables and assuming homogeneity within the sample. Fewer studies have applied person-centered approaches like latent class analysis (LCA) or latent profile analysis (LPA), differentiated by the former examining categorical variables and the latter continuous variables. Person-centered analyses are based on underlying (latent) classes or subgroups, and these characteristics may be present as meaningful patterns or profiles (14); here the most likely profile membership of each youth is estimated (15). Person-centered analyses, such as LPA and LCA, are particularly helpful for the identification of possible similarities and dissimilarities across several internalizing symptoms in a heterogenous sample.

Only a few studies have conducted LPA/LCA on internalizing symptom subtypes and profiles (16); these have found that the profiles differ mainly on symptom levels rather than the shape of the symptom profile (17, 18). One exception is the LCA study of Burstein et al. (19) of sex- and age-specific structures and comorbidity in lifetime anxiety disorders among US adolescents (*N* = 2,539; *M* = 15.2 years; range, 13–18). The authors identified classes of anxiety subtypes, where one specific anxiety subtype (e.g., separation anxiety or social phobia) peaked in symptom level among youth in a population-based sample. In the same study, they found few classes of multiple anxiety disorders consisting of same-level symptoms. These latter classes were associated with more clinical severity, impairment, and comorbidity; sex differences were found in a variety of anxiety subtypes but not across age groups (19). The study of Burstein et al. (19) focused on lifetime anxiety disorders; however, retrospective responses may have influenced their findings. Furthermore, other studies using LCA on general adolescent samples mainly find anxiety and depression symptoms with profiles differentiated by level of symptoms, such as low, moderate, and high/severe symptom profiles (17, 20). Clinically referred children and youth have been found to have higher levels of internalizing symptoms compared to non-referred peers in the general sample (20). Nevertheless, these studies are limited to general population youth samples or compare a clinical with a general population youth sample; studies including samples of youth in foster care are therefore lacking.

To our knowledge, no other studies have applied LPA on internalizing symptoms among youth in foster care, compared to clinical and general population youth samples. There is reason to believe that youth in foster care have a higher likelihood of belonging to profiles with a higher symptom level of internalizing symptoms compared to same-age general population youth samples. However, it is less certain as to whether they have a higher likelihood of belonging to specific symptom profiles compared to same-age clinical youth samples. Youth in foster care have often experienced several out-of-home care placements (21) and might have multiple maltreatment experiences prior to placement (22). Hence, compared to their clinical peers, youth in foster care are more likely to have been exposed to parental psychopathology and negative parenting behaviors, both associated with increased risk of child psychopathology (23). Furthermore, adverse care experiences are one of the risk factors shared between depression and anxiety (24). This raises the question of whether youth in foster care run a higher risk of belonging to specific profiles of internalizing symptoms compared to their non-placed peers.

Various studies have identified possible mechanisms for the associations between maltreatment and anxiety—for example, changes in brain functioning (25), neuropsychological functioning (26), sleep (27), and cognition (28). These studies do not explore differences in anxiety subtypes. However, an increase of certain anxiety symptom subtypes could be expected among foster youth compared to a general population youth sample. A previous study of youth in foster care found that social and generalized anxiety were the most frequent anxiety symptom subtypes. Exposure to sexual abuse was the only maltreatment type associated with an increase in the level of all anxiety symptom subtypes, whereas neglect yielded increased symptom levels for all anxiety subtypes, except separation anxiety and fear of physical injury (i.e., simple phobia). Physical/emotional abuse increased the symptom level for social anxiety and OCD (29). These findings indicate the relevance of investigating subtypes of anxiety, as this may be affected by the specific maltreatment experiences of the youth and manifest itself in the shape of the symptom profile for foster youth. Increased knowledge on these matters is also needed to discuss potential adjustments and improvements in interventions and treatment programs offered to youth in foster care.

If youth in foster care are more likely (1) to be comparable to a general population youth sample and (2) belong to a high symptom profile, one may hypothesize that interventions targeting internalizing problems work for all youth across care conditions. However, if clear group differences appear, standard interventions may need to be tailored to the individual needs of youth in foster care.

In summary, knowledge is lacking regarding the level and pattern of internalizing symptom subtypes among youth in foster care compared to their peers in clinical settings and in the general population.

The main objectives of this study are 2-fold: first, to examine the symptom profiles of self-reported anxiety and depression across three unique samples (youth in foster care, youth in clinical treatment for internalizing problems, and a general population youth sample); and second, we investigated whether these symptom profiles were predicted by affiliation to group (i.e., their specific sample) and age and whether there were potential interactions of sex.

## Materials and Methods

This study used data from three unique samples investigating internalizing symptoms in youth: a foster care sample (30), a clinical sample (31), and a general population youth sample (32). The total sample consisted of 2,005 youth (53.1% girls) with a mean age of 13.96 years (SD = 1.19; range, 11–18).

### Participants and Procedures

#### Foster Care Sample

The foster care sample (*N* = 303, 41.9% response rate) was drawn from the second wave of the longitudinal cohort study Young in Foster Care (hereafter the YIF study). Youth were recruited between October 1, 2016 and March 31, 2017, from the southeastern region of Norway. Eligibility was considered through the regional records from the Office for Children, Youth, and Family Affairs South (*n* = 573) and through municipal child-protective services (*n* = 279). Background information for all eligible youth were collected from the head of each municipal child-protective services. Information letters and an invitation to participate were sent by postal mail. Reminders were sent by post and subsequent telephone contact. Youth eligible to participate were born between 1999 and 2005 and had lived in their current foster care home for at least 6 months. A total of 724 eligible youth were included, out of which 303 completed the Strength and Difficulties Questionnaire (SDQ; 41.9% response rate), comprising the total sample in the present study. The SDQ was used to screen for anxiety problems. An affirmative response on at least one item on the SDQ emotional subscale qualified as completing the assessment for anxiety symptoms. The mean age was 14.82 years (SD = 2.04, range 11–18), and 46.5% were girls. The participating youth completed questionnaires, either online on a secure website or by phone interview. All participating youth received a gift card approximately EUR 31 (NOK 300).

#### Clinical Sample

The clinical sample comprised the child-focused part of the Assessment and Treatment—Anxiety in Children and Adults study (hereafter the ATACA study; *N* = 182). This was a randomized controlled trial evaluating the effectiveness of group and individual cognitive behavioral treatment for anxiety disorders in seven child and adolescence community mental health clinics in Norway. The participants were recruited between January 1, 2008 and April 30, 2010. The study included 8–15-year-olds; however, in the present study, we included only those between 11 and 15 years. The mean age was 12.82 years [SD = 1.42, range 11–15; for further details on the participants and procedure, see Wergeland et al. (31)]. In the present study, baseline data from the self-report of youth on the Spence Children's Anxiety Scale and the Short Mood and Feelings Questionnaire were included.

#### General Population Youth Sample

The general population youth sample is population-based and was collected in a school survey conducted in 18 schools in 10 municipalities in the east, west, and south of Norway between October 15, 2014 and March 11, 2015. This was part of the Low-Intensity vs. Standard Cognitive Behavioral Therapy for Anxious Youth study (hereafter the LIST study). The school survey comprised of 1,719 youth [mean age, 13.91 years; SD = 0.86; range, 12–16; 51.4% girls; for further details on the participants and procedures, see Raknes et al. (33)]. In the present study, the self-report of youths on the Spence Children's Anxiety Scale and the Short Mood and Feelings Questionnaire was included.

### Ethics

The Regional Committee for Medical and Health Research Ethics, Western Norway, approved the three studies (YIF 2010/2367-1, ATACA 2011/1297; and LIST 2013/2331). Consistent with Norwegian legislation, youth in foster care aged 11–15 years were invited through letters addressed to the foster parents to first obtain the consent of their carers; these youth then gave their consent by completing the questionnaire. Youth aged 16 and older were approached directly by postal mail with an invitation and information; they consented to participation on their own behalf. The Norwegian Directorate for Children, Youth, and Family Affairs provided exemptions from confidentiality for each municipal child-protective services. For the ATACA and LIST studies, written informed consent was obtained from the parents and from the youth themselves (31, 32).

### Measures

#### Sociodemographic Information

In the YIF study, the sex and age of the youths were acquired through the regional records of the Southern Regional Office for Children Youth and Family Affairs and confirmed in interviews with the municipal child-protective services. In the ATACA study, data on sex and age were collected from the web-based diagnostic interview, Development and Well-Being Assessment (34), completed by the parents; in the LIST study, these were collected through a questionnaire administered at the schools during school hours and completed by the youth.

#### Depressive Symptoms

Depressive symptoms were measured with the Short Mood and Feelings Questionnaire, child version (SMFQ-c) (35). The SMFQ-c consists of 13 items rated on a three-point scale (0 = “not true” to 2 = “true”), generating a total scale score of 26. The SMFQ-c has good construct validity, converging validity, and reliability (36–38). The internal consistency of the SMFQ-c in the present sample was excellent (Cronbach's α = 0.91).

#### Anxiety Symptoms

Anxiety symptoms were assessed with the Spence Children's Anxiety Scale, child version (SCAS-c) (39). The SCAS-c consists of 44 items, including six positive filler items, rated on a four-point scale (0 = “never” to 3 = “always”), with a maximum score of 114. The SCAS-c comprises six subscales of anxiety coinciding with the anxiety disorders in the DSM-IV: “separation anxiety,” “social anxiety,” “obsessive–compulsive disorder” (OCD), “panic/agoraphobia,” “physical injury fears,” and “generalized anxiety.” The psychometric properties of SCAS-c are considered robust (40). The internal consistency for the total scale score of SCAS-c in the present sample was excellent (α = 0.89). Except for the physical injury fears subscale (α = 0.57), the internal consistency of all subscales ranged from acceptable to excellent: separation anxiety (α = 0.74), social anxiety (α = 0.79), OCD (α = 0.75), panic/agoraphobia (α = 0.85), and generalized anxiety (α = 0.83). The mean score of each subscale was used in the analysis of the present study, with a range of 0–3.

#### Missing Data Across Measures

Youth who had responded to both the SCAS-c and SMFQ-c in the respective studies were included in the present analysis. This means that 66 youth (3.2%) were excluded from the general population youth sample, 25 (1.2%) were excluded from the clinical sample, and 58 (2.8%) were excluded from the foster care sample. After exclusion, the total number was 2,055 youth. Due to differences in the age range in each of the subsamples, the age cutoff was set at 11 and 18 years; this resulted in the exclusion of 50 participants. No participants were excluded based on sex or age, as they all answered these questions. The total included sample size was thus 2,005 youth.

### Statistical Analysis

Descriptive statistics were reported for each of the samples using IBM SPSS Statistics for Windows, version 25.0 (41). The LPA was conducted with Mplus, version 8.0 (14). We conducted a three-step LPA to explore whether there were profile patterns based on the anxiety symptom subtypes (e.g., social anxiety or generalized anxiety) and depressive symptoms in the total sample across the three samples (42–44). We tested one to six profile solutions. The following fit indices and their conditions were considered for the best model fit: lower values on Akaike's information criterion (AIC), Bayesian information criterion (BIC), and sample-size adjusted BIC (SABIC) (45). A scree plot was used to identify a curve or “elbow” for lowered AIC and BIC values (46). An insignificant Lo-Mendell–Rubin (LMR) likelihood ratio test and a bootstrap likelihood ratio test (BLRT) for each added class gave support to the fewer class solution. An entropy value ~1 was considered more beneficial for a more defined classification of the participants (42). Given our aim to identify unique symptom profiles across the three sub-samples, we were not solely interested in the best-fit indices for the total sample: we sought instead to detect qualitatively different profiles, which differed in shape and level. The shape of the symptom profile can be explained as the pattern that constitutes the total increases and decreases of specific points in the plot of anxiety symptom subtypes and depression symptoms.

Following the default in Mplus, variance was set to equal and covariances to zero. As required, to exclude the local maxima, the best log-likelihood value was replicated and increased twice the random start (1,000 250/2,000 500), in which the best log-likelihood was still obtained and replicated. The number of iterations amounted to 500.

The covariates sex, age, and dummy-coded group affiliation (foster care, clinical, or general population youth sample, of which either the general or clinical sample functioned as a reference group) were included in the three-step analysis. Age was mean-centered in all the analyses. As part of the three-step LPA, multinominal logistic regression was used to test whether the covariates predicted the latent profile membership. Interaction analyses were conducted to investigate the effect of sex on group affiliation. The estimated probabilities of 11–18-year-old youth (the mean age) in the general, foster care, and clinical populations were calculated from the results of the multinominal regression analyses.

## Results

### Fit Indices and Model Retention Decision

**Figure 1:**
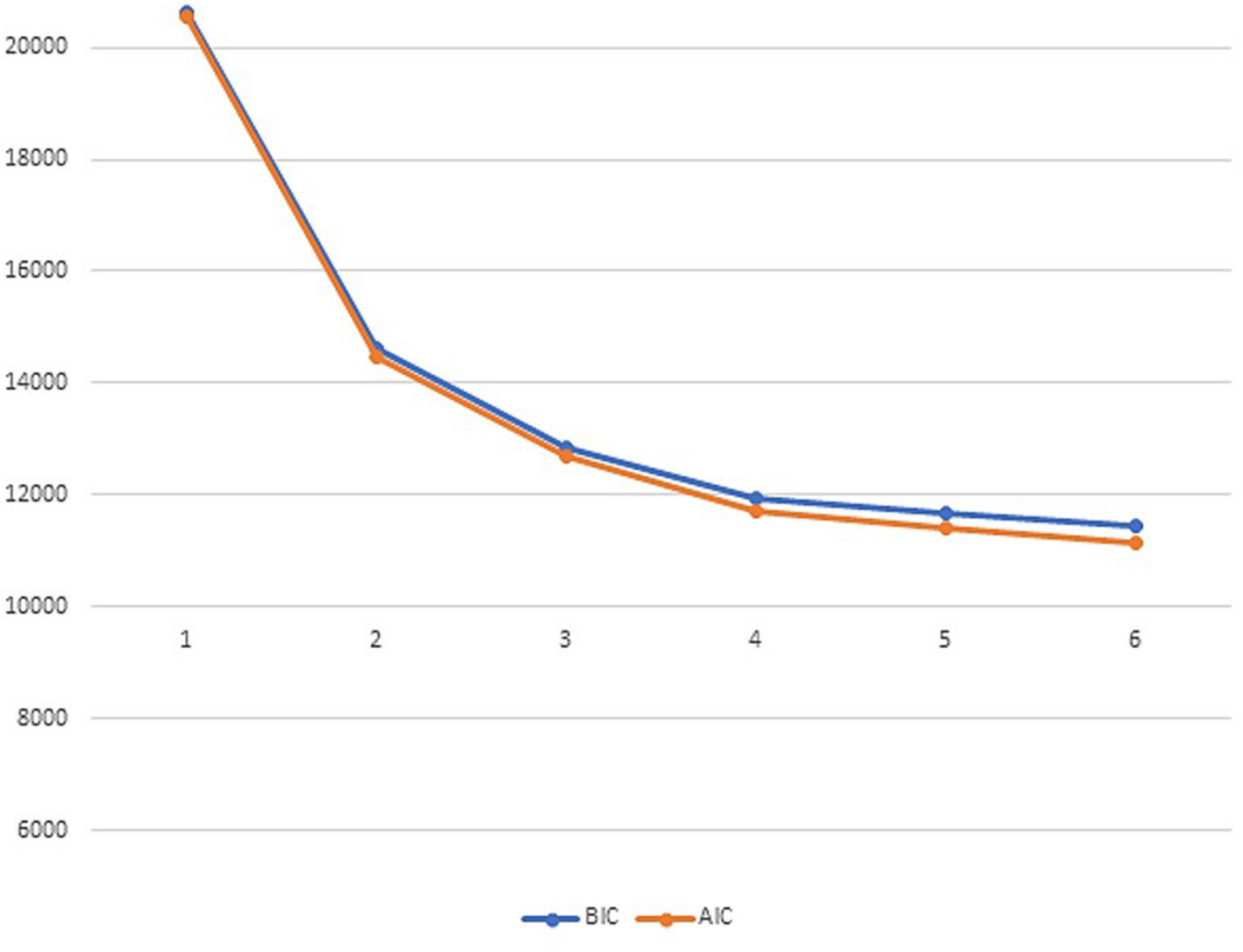
Scree plot for AIC and BIC for six latent profiles of self-reported anxiety and depressive symptoms across a foster care, clinical, and general population youth samples. The scree plot is based on the latent profile analysis of one to six profile solutions. AIC, Akaike's information criterion; BIC, Bayesian information criterion.

**Figure 2:**
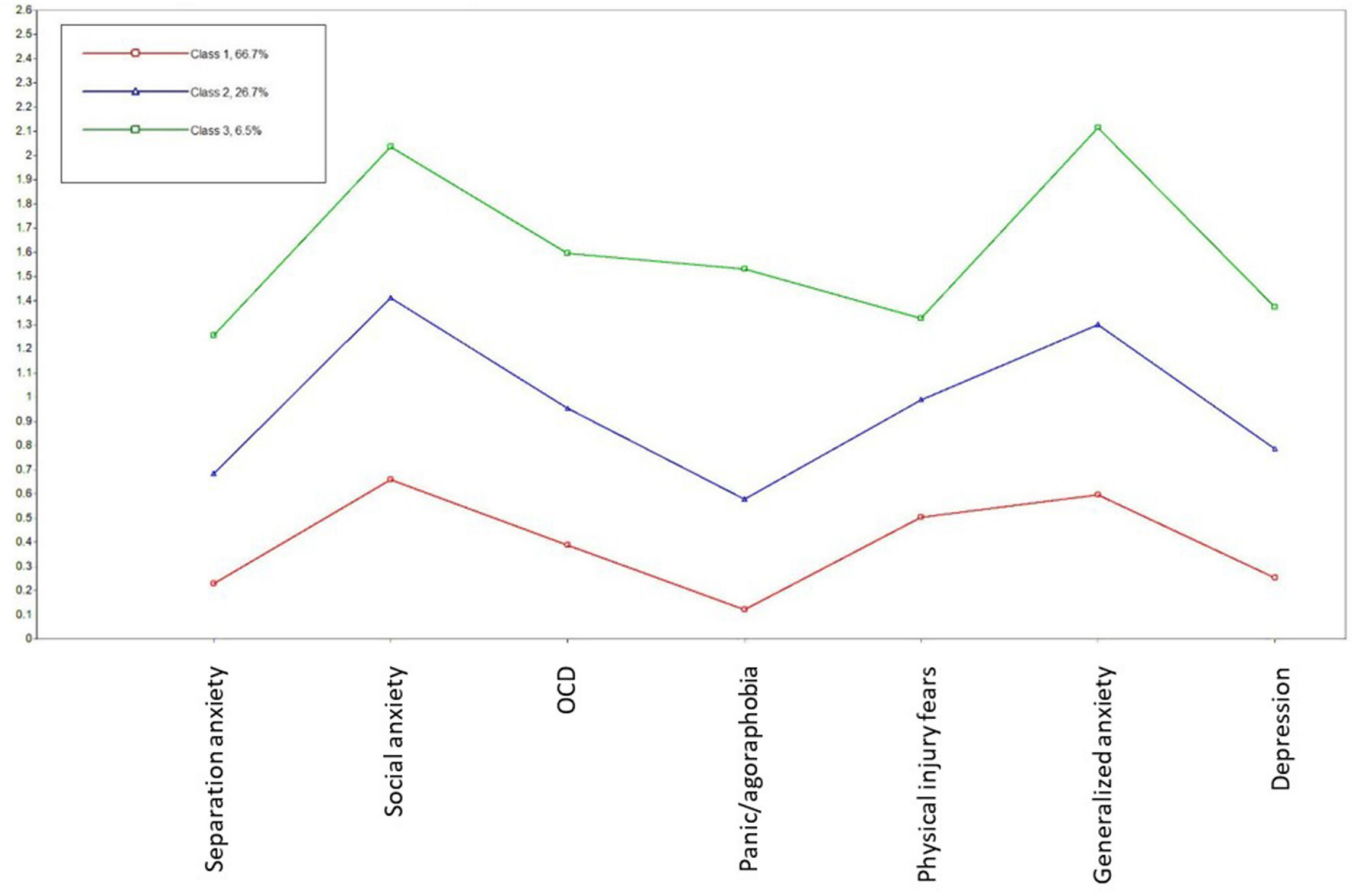
Three latent profiles of internalizing symptoms across a foster care, clinical, and general population youth samples. Low symptom profile, *n* = 1,366; medium symptom profile, *n* = 507; high symptom profile, *n* = 120.

**Figure 3:**
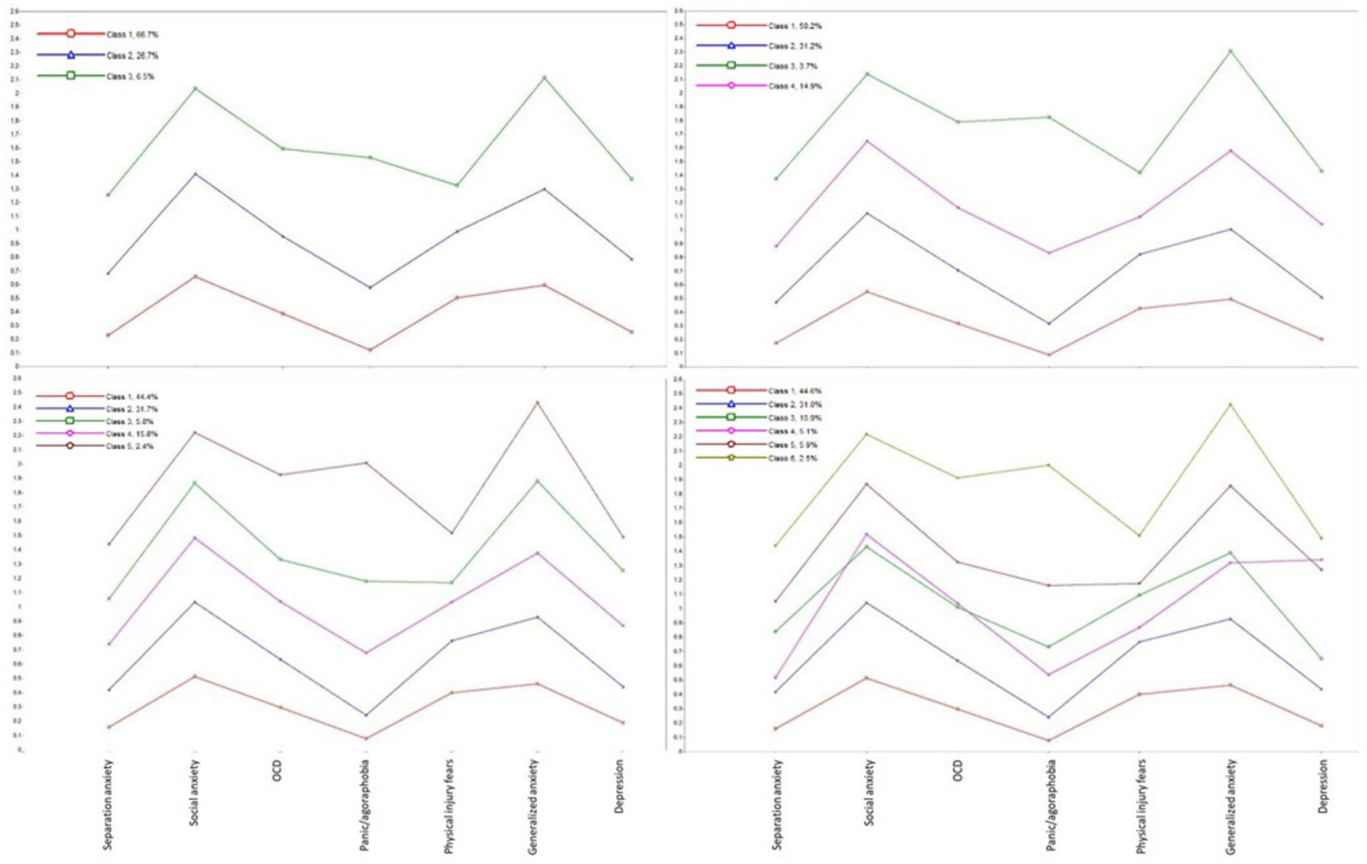
Three, four, five, and six latent profiles on internalizing symptoms across a foster care, clinical, and general population youth samples. From the top-left corner: three-symptom profile (final model solution) and four-symptom profile. From the bottom-left corner: five-symptom profile and six-symptom profile. The internalizing symptoms are represented at the bottom two profiles as separation anxiety, social anxiety, obsessive–compulsive disorder, panic/agoraphobia, physical injury fears, generalized anxiety, and depression symptoms.

Table 1 presents the descriptive statistics for all three youth samples separately and as a total sample. Table 2 shows the fit indices for the LPA model from one to six profiles. The fit indices did not present one clear finding for the best model fit. The AIC, BIC, SABIC, and BLRT supported a six-profile solution. The LMR, however, supported a four-profile solution as it did not have a statistically significant poorer fit than the five-profile solution. The four-profile solution was also supported by a scree plot (Figure 1), in which there was an “elbow” of the curve (47) for this profile solution. However, we decided on the three-profile solution (see Figure 2) as the most parsimonious, as a visual inspection of the profile plots revealed that adding more profiles above three mainly affected the level of the profiles and not their shape (see Figure 3 for the three-, four-, five-, and six-profile solutions). Moreover, in the four-, five- and six-profile solutions, the smallest class consisted of fewer than 5% of the participants, which could be a sign of an overextracted and unstable class solution (47).

**Table 1:** Descriptive statistics for the foster care, clinical, and general population youth samples.

**Sample**	***N***	**Girls, % (*n*)**	**Mean age (SD)**	**Range**
Foster care sample	245	51.4 (126)	14.85 (2.08)	11–18
Clinical youth sample	107	59.8 (64)	12.82 (1.42)	11–15
General youth population	1,653	52.9 (875)	13.91 (0.86)	12–17
Total sample (after exclusion)	2,005	1,065 (53.1)	13.96 (1.19)	11–18

*The table shows the final number of youths included in the present study. Youth from each of the three samples were included only if they had responded to both measures of anxiety and depression*.

**Table 2:** Model fit summary of the latent profile analysis of the foster care, clinical, and general population youth samples.

**Model**	**LL**	**AIC**	**BIC**	**SABIC**	**Entropy**	**LMR *p*-value**	**BLRT *p*-value**
1	−10,251.604	20,531.208	20,609.656	20,565.177			
2	−7,220.769	14,485.537	14,608.812	14,538.917	0.942	0.0000	*p* <0.0001
3	−6,314.862	12,689.725	12,857.827	12,762.515	0.922	0.0214	*p* <0.0001
4	−5,827.814	11,731.628	11,944.557	11,823.829	0.874	0.0018	*p* <0.0001
5	−5,661.635	11,415.270	11,673.027	11,526.882	0.856	0.1355	*p* <0.0001
6	−5,528.210	11,164.421	11,467.005	11,295.443	0.864	0.0085	*p* <0.0001

*N = 2,005.*

*LL, log-likelihood; AIC, Akaike's Information Criterion; BIC, Bayesian Information Criterion; SABIC, sample size-adjusted BIC; LMR, Lo-Mendell–Rubin; BLRT, bootstrap likelihood ratio test*.

### Symptom Profile Representation

The plot of the three-profile solution is shown in Figure 2. The mean levels of the symptoms within each of the three latent profiles are displayed in Table 3. The three symptom profiles were named as follows: (i) low symptoms (66.7% of the sample), (ii) medium symptoms (26.7%), and (iii) high symptoms (6.5%). Besides the general increase in symptom level, there were some differences in shape for the low, medium, and high symptom profiles. The medium symptom profile differed in shape from the low symptom profile by having a more profound increase in social anxiety and generalized anxiety symptoms relative to the other internalizing symptoms. The shape of the high symptom profile distinguished itself from the other profiles by having a particularly high increase in panic/agoraphobia and generalized anxiety symptoms and a relatively low increase in physical injury fears relative to the increase in other internalizing symptoms.

**Table 3:** Mean scale scores for the three latent symptom profiles.

**Variable**	**Low symptoms**	**Medium symptoms**	**High symptoms**
	***n* = 1,338**	***n* = 536**	***n* = 131**
Separation anxiety	0.228 (0.016)	0.683 (0.038)	1.256 (0.073)
Social anxiety	0.660 (0.027)	1.412 (0.054)	2.036 (0.058)
Obsessive–compulsive disorder (OCD)	0.389 (0.017)	0.955 (0.050)	1.596 (0.081)
Panic/agoraphobia	0.121 (0.007)	0.579 (0.057)	1.530 (0.122)
Physical injury fears	0.504 (0.020)	0.988 (0.035)	1.325 (0.067)
Generalized anxiety	0.596 (0.023)	1.301 (0.055)	2.115 (0.078)
Depression symptoms	0.253 (0.013)	0.786 (0.061)	1.372 (0.042)

*Results are reported as mean levels and standard errors in parentheses. Means and standard errors for variables across all profiles: separation anxiety, M = 0.43 (SD = 0.45); social anxiety, M = 0.94 (SD = 0.60); OCD, M = 0.63 (SD = 0.50); panic/agoraphobia, M = 0.35 (SD = 0.46); physical injury fears, M = 0.69 (SD = 0.54); generalized anxiety, M = 0.88 (SD = 0.58); and depression symptoms, M = 0.47 (SD = 0.46). The symptoms of anxiety were measured with the Spence Children's Anxiety Scale—Child. The symptoms of depression were measured with the Short Mood and Feelings Questionnaire—Child*.

### Associations Between Symptom Profiles and Age, Sex, and Group Affiliation

**Figure 4:**
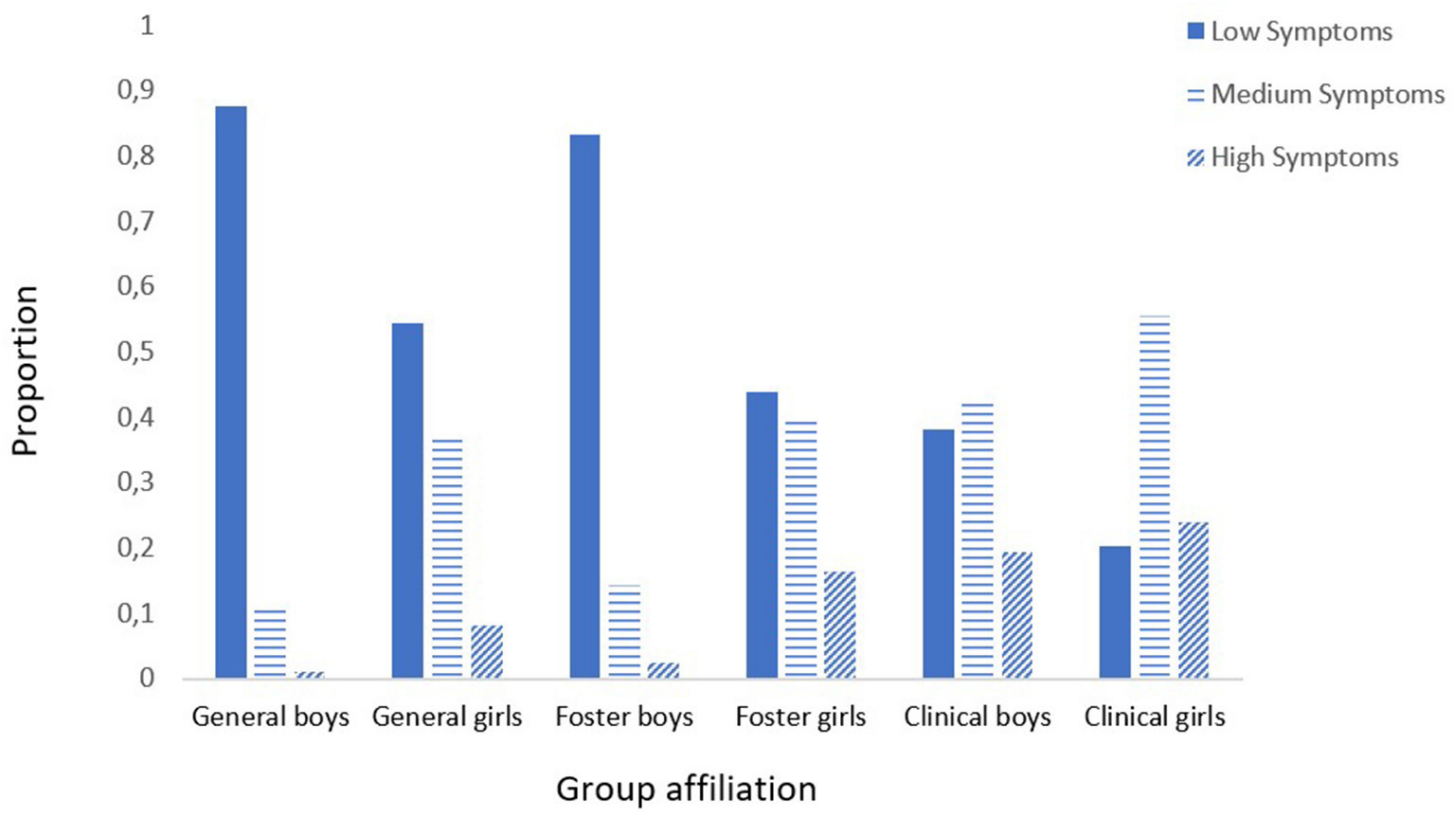
Proportion of boys and girls in the foster care sample (*n* = 245), the clinical sample (*n* = 107), and the general population youth sample (*n* = 1,653) and the estimated distribution into low, medium, and high symptom profiles. The figure shows the proportion of girls and boys in the foster care, clinical, and general population youth sample estimated to belong to the three-symptom profile. The figure is based on the interaction analysis between sex and the clinical youth sample. The analysis was controlled for the mean age for all three samples.

Age, sex, and group affiliation (with the general population youth sample as the reference group) predicted the probability of medium or high profile membership relative to the low symptom profile. The interaction effect between sex and the clinical sample, as well as sex and the foster care sample, was included. The sex by clinical sample interaction was significant (β = −1.756, *p* = 0.015), but the sex by foster care sample interaction was not (β = 0.198, *p* = 0.765). The estimated probabilities of youth being in the low, medium, or high symptom profile) depending on group affiliation are presented in Figure 4.

#### Medium Symptoms vs. Low Symptoms

Youth in the clinical sample (β = 2.154, *p* < 0.001), but not the foster care sample (β = 0.284, *p* = 0.140), had a higher probability of belonging to the medium (vs. low) symptom profile when compared to the general population youth sample (see Table 4). Youth in the clinical sample also had a higher likelihood (β = 1.87, *p* < 0.001) of belonging to the medium (vs. low) symptom profile when compared to youth in foster care. Furthermore, belongingness to the medium symptom profile was predicted by being a girl (β = 1.665, *p* < 0.001), but not by age (β = 0.071, *p* = 0.196).

**Table 4:** Anxiety and depression symptom profile representation among youth in foster care (*n* = 245) and clinical youth (*n* = 107) compared to a general population youth sample (*n* = 1,653).

	**Medium symptoms**	**High symptoms**
	***n* = 536**	***n* = 131**
	**β (SE)**	**β (SE)**
Foster care sample	0.284 (0.19)	*0.911 (0.283)*
Clinical sample	*2.154 (0.393)*	*3.820 (0.606)*

*The general population youth sample (n = 1,338) served as the reference class. The results are reported in logits. The symptoms of anxiety were measured with Spence Children's Anxiety Scale—Child. The symptoms of depression were measured with the Short Mood and Feelings Questionnaire—Child. Statistically significant results are marked in italics.*

*β, standardized beta; SE, standard error of the standardized beta*.

#### High Symptoms vs. Low Symptoms

Compared to youth in the general population sample, youth in foster care had an increased risk of belonging to the high (vs. low) symptom profile (β = 0.911, *p* < 0.001; see Table 4). The clinical sample had a higher likelihood of belonging to the high symptom profile when compared to the general population youth sample (β = 3.820, *p* < 0.001) and also when compared to the foster care sample (β = 2.909, *p* < 0.001). Girls had an increased risk of belonging to the high (vs. low) symptom profile (β = 2.595, *p* < 0.001). The effect of the two predictors of clinical youth sample by sex significantly interacted with each other (β = −1.756, *p* = 0.015). As shown in Figure 4, the effect of sex was nearly erased in the clinical sample. In contrast to the general and foster care sample, girls, and boys in the clinical sample were both estimated to belong to the high symptom profile. Finally, increasing age was associated with a higher likelihood of being in the high symptom profile (β = 0.322, *p* < 0.001).

## Discussion

This study investigated the symptom profiles of anxiety and depression in three samples of 11–18-year-olds: youth in foster care, youth clinically referred to specialized mental health services for treatment of anxiety and depression, and a general population youth sample. By using LPA, we identified three distinct profiles: one with low, one with medium, and one with high levels of internalizing symptoms, which also differed somewhat in shape. The latent symptom profiles were predicted by group affiliation: that is, when compared to the general population youth sample, youth in foster care were more likely to belong to the high symptom profile. Youth in the clinical sample were more likely to belong to the medium and high symptom profiles compared to youth in both the general population youth sample and the foster care sample. Furthermore, sex and age were related to symptom profiles, with girls being more likely to belong to the high symptom profile. The likelihood of having higher symptoms increased with age.

Our finding of three symptom profiles differentiated between low, medium, and high is consistent with previous findings: mainly that there are differences in levels rather than the shape or pattern of internalizing symptoms. Wadsworth et al. (20), for example, identified three levels of symptom profiles of anxiety and depression among both non-referred and clinically adolescents (20). Furthermore, van Lang et al. (17) found that groups of adolescents with both anxiety and depression had high, moderate, and low symptom levels (17). Others have also concluded that high levels of one specific type of anxiety were generally not concurrent with low levels of other anxiety disorders (18, 48).

In the present study, we found that youth in foster care reported high symptom levels compared to general youth; however, compared to clinical youth, they showed an overall lower symptom load (Table 4). Youth in the general population sample were identified as more likely to belong to the low symptom profile and the clinical youth sample to the high symptom profile. This is in line with other studies in which youth in general populations have been found to have lower symptoms of anxiety and depression and youth clinically referred with internalizing problems have higher symptom levels (20, 49). As noted earlier, however, similar studies on youth in foster care are lacking.

Wadsworth et al. (20) reported a smaller gap between the sexes in pre-adolescent years for anxiety and depression problems, while in adolescence this gap increased. Other studies have confirmed increased symptoms of depression (50) and certain anxiety subtypes such as generalized anxiety and social anxiety during adolescence (49). This is in line with our findings that internalizing symptom levels increased with older age.

Our finding that youth in foster care are more likely to belong in the high symptom profile compared to the general population youth sample strengthens the argument that internalized symptoms in youth entering foster care should be assessed. While elevated levels of internalizing symptoms may reflect a history of maltreatment and relational problems among youth in foster care (29), these may also reflect how demanding the transition into a new home may be for these youth. As they must establish new relationships away from their primary carers (51, 52), these challenges may trigger or maintain symptoms of anxiety or depression in youth in foster care. The development of internalizing problems in youths should also be viewed within the caregiving context. Several aspects should be considered here. Placement instability is often a reported concern, as it may contribute to disrupting the developmental growth and resilience in youths (53). Through the experiences of safe and caring adults, youths will strengthen their relational abilities in forming new relationships with other adults and peers. As youth in foster care often have a background of maltreatment, adapted family interventions may be necessary. The supervision of foster parents, focusing especially on how to provide care for children and youth who independently do not seek this, is found effective in reducing internalizing problems among children and youth in foster care (54).

Even when high levels of internalizing symptoms are reported, youth in foster care are often not referred to Child and Adolescent Mental Health Services (CAMHS) (30). This may be due to internalizing problems often being more concealed compared to other mental health symptoms in youth, for example, externalizing problems. Thus, these may be difficult for caregivers, caseworkers, or health care practitioners to detect. Factors within the youth themselves may also hinder their anxiety or depression from being noticed—for example, youth may lack mental health literacy or see their long-term mental health symptoms as normal. Furthermore, coping with other life challenges—like a new foster home where they must get to know, trust, and build a relationship with foster parents and other family members—may feel more imperative than their mental health challenges (53, 54). Research has also identified barriers for help-seeking behaviors among young people, in general, which include fear of stigma and preference for self-reliance (55). These are barriers that might be especially relevant for youth in foster care due to care experiences that increase the risk of a reserved relational strategy such as impaired ability to seek and accept comfort (22). Youth in foster care may therefore benefit from an initial screening process for internalizing symptoms.

Our findings regarding sex differences in symptom levels among youth in the clinical sample are in line with other studies showing that girls in clinical samples report more internalizing problems than boys (19, 20). In the foster care sample, sex differences were not found for internalizing problems during childhood [6–12 years (12)] but seem to have emerged during their teenage years as found in the present study. These findings are consistent with studies on general and clinical youth populations confirming that, with increasing age, sex differences appear in the prevalence of internalizing problems (56–58). Furthermore, in the present study, girls in foster care were more likely to have medium or high symptom profiles compared to boys in both the general population sample and in the foster care sample. Girls in foster care also seemed to have a profile more similar to clinically referred boys in that both groups were more inclined to belong to the high symptom profile. However, boys in the general and foster care samples seemed to have higher probabilities of belonging to the low symptom profile. These findings indicate that there are important differences in symptom load across a specter of internalizing problems depending on sex among youth in foster care.

Our findings have implications for what the dominant focus of mental health services and foster parents should be. Regarding internalizing symptoms, a thorough assessment of girls in foster care is needed. A previous study reported sex differences in maltreatment exposures such as physical/emotional abuse, neglect, and sexual abuse among youth in foster care, with girls being more exposed (29). Furthermore, in this same study, girls in foster care were found to have higher anxiety symptoms when subjected to all three maltreatment types and higher depression symptoms when exposed to neglect compared to boys. This may indicate a greater vulnerability to internalizing symptoms among girls in foster care compared to boys (29).

Although we find clear differences regarding the levels of symptom profiles, fewer differences in the shape of the symptom profiles were identified. According to Dozois et al. (24), the psychopathology of parents seems to be a risk factor affecting a broad range of internalizing problems. This is related to our previous findings where different maltreatment types were associated with a broad range of internalizing symptoms (29). These findings may partly explain why, in the present study, we do not identify profiles with large differences in shape and that youth in foster care did not have a higher risk of belonging to a specific symptom profile compared to their peers in the clinical and population-based samples.

The most profound difference between the shapes of the three profiles, however, is the increase in panic/agoraphobia symptoms in the high symptom profile, whereas in the low and medium symptom profiles panic/agoraphobia was the rarest anxiety symptom subtype of anxiety. One might speculate that panic attacks may be more easily triggered by the other anxiety subtypes—e.g., agoraphobia, separation anxiety, and social anxiety—if the level is high enough. Panic attacks are typically characterized by incidents of intense fear and are accompanied by intense physiological symptoms (like heavy breathing, lightheadedness, and increased pulse) as well as cognitions like one is “about to die” or “become insane” (43). Research shows that impairing symptoms lead to clinical treatment, possibly due to the attention these receive from the caregivers of youth or other significant adults (44). Other studies show that panic attacks relate to help-seeking behavior due to the impairment they cause (45, 46): this may explain why the clinical youth had a higher likelihood of belonging to the high symptom profile, which has a higher panic/agoraphobia than the low symptom profile, to which youth from the general population sample are more likely to belong.

In the present study, the symptom subtype of generalized anxiety also had a relatively more pronounced increase in the high symptom profile of internalizing symptoms. Generalized anxiety covers a range of indiscriminate fears, where even worrying may be believed to be dangerous (47). It is also associated with physiological symptoms like headaches, muscle tension, and sleep problems over an extended period (43). These symptoms may influence important life areas such as school achievements and social relations. If this is the case, the symptoms may be impairing and may be a prioritized condition for admission to the CAMHS. However, whether panic and generalized anxiety are more impairing than other internalizing sub-scales (e.g., social anxiety or depressive symptoms) is not clear and needs further examination.

In the present study, sex and group affiliation predicted three symptom levels (low, medium, and high symptom profiles) across subtypes of internalizing symptoms. However, in line with Burstein et al. (19), we found a “complex” profile (our high symptom profile) to which girls in foster care and youth in clinical treatment had a higher probability of belonging. The present findings emphasize that girls, compared to boys, in foster care have a relatively high symptom load, but without major spikes in specific subtypes of internalizing symptoms. Moreover, their levels of depressive symptoms follow their levels of anxiety symptom subtypes and do not seem to be separate from the different anxiety symptom subtypes. This is in line with research indicating that anxiety is a predictor of depression (59, 60). It may seem that those suffering from internalizing symptoms have overall high symptom levels across subtypes, also including depressive symptoms. The finding that levels of anxiety and depressive symptoms follow each other in terms of symptom load across the three profiles may indicate that a transdiagnostic understanding of internalizing problems is important even if the profiles differed somewhat in shape (24, 61).

## Strengths and Limitations

A strength of the present study is its comparison of three unique youth samples: a foster care sample, a general population youth sample, and a clinical sample. Each of the three studies, from which the sample of the present study was drawn, had methodological strengths that offer insights into the expression of youth anxiety and depressive symptoms. The total sample is large and heterogenous, generating solid results across different groups of youth, and with sufficient variance to run LPA. The total sample consists of youth who have completed identical measures of anxiety and depressive symptoms, with well-documented psychometric properties. Methodologically, LPA has the advantage of identifying latent profiles across groups and classifying the probability of membership of youth in these profiles compared to the others.

Although the present study has important strengths, it also has some limitations. One limitation concerns the final model decision. Although the fit indices pointed toward a four-profile solution, we decided on a three-profile solution. This was justified through our aims of choosing a model with profiles unique in their shape *and* level. Our decision was somewhat subjective as the fit indices were not consistent, and we concluded that adding more profiles would only lead to differences in level and not shape. However, our choice of a three-profile solution is also supported. The four-, five- and six-profile plots are shown in Figure 3 and reveal that increasing profiles almost exclusively contribute with changes in level. In the four-, five-, and six-profile solutions, the smallest classes consist of fewer than 5% of the participants, which is not considered ideal (47). Furthermore, the age range of the total sample (11–18) may be considered too wide and could possibly affect the analyses. We tested this by including only youth between ages 11–15 years old across the three samples in the LPA. However, the results were very similar and thus did not impact the results.

Another limitation is the difference in time points for the three periods of data collection: in particular, the large time difference between the assessment of the foster care sample (2016–2017) and the clinical sample (2008–2010). Possible time trends and events of social significance may impact the mental health of young people. Indeed levels of internalizing problems seem to have increased (especially for older girls) in the 21st century, worldwide (62), and in Norway (63, 64). Norwegian youth may have been influenced by the financial crisis in 2008, thus affecting the scores of the clinical sample; however, research indicates varying results regarding associations between socioeconomic status and mental health problems among youth in Norway (64). It is also possible that the internalizing symptom levels in the foster youth and the general population youth sample could have been influenced by the terrorist attacks in Oslo and Utøya in 2011: these seemed to particularly affect the mental health of Norwegian youth who had experienced prior adversity (65, 66).

A final limitation is that the response rate for the general population youth sample was relatively low.

## Clinical Implications

The elevated risk among youth in foster care, particularly girls, to belong in the high-level profile of internalizing symptoms indicates that health services and child-protective services should be alert to the presence of internalizing symptoms and offer treatment when necessary. However, being in foster care *per se* is not sufficient to warrant access to assessment and treatment for internalizing disorders. An early-step care approach, with screening as a first step, could identify youths in foster care in need of more thorough assessment and treatment.

As at-risk youth may suffer more privately of internalizing symptoms, lacking the stability of concern of a caregiver, standardized early assessments and interventions may be even more crucial for children and youth in foster care. Timely assessment is essential to identify possible risk factors in the characteristics of a child (e.g., temperamental inhibition) and history (e.g., maltreatment exposures) as well as parental rearing styles and other factors conceivably maintaining the internalizing problems into adulthood (67). Considering that youth are in a critical neurobiological and socio-developmental phase in life, this may be a pivotal time for impacting further trajectories of internalizing problems (68).

An early assessment of risk factors, following the progression of the internalizing problems over time, could help the clinician to offer targeted effective interventions to the current stage of problems as early as possible (69). In a framework of a clinical staging model, clinicians integrate the biological, social, and contextual aspects of youth into their clinical and diagnostic understanding of the youth and how these factors may impact their problems (70). The treatment and interventions are consequently more personalized to the specific needs of the youth (71). A first international consensus statement for transdiagnostic clinical staging for youth mental health is proposed, stating its principles and assumptions (72). Although promising, the adoption and evaluation of such a framework remains to be seen for the foster care population, as this group is not automatically referred to mental health services.

However, it is unknown if youth in foster care with internalizing problems benefit from adjusted treatment approaches. Possible adjustments could be content-wise, for instance, by added focus on treating trauma and stress-related symptoms or enhancing specific skills such as problem-solving, relational functioning, or augmentation of specific procedures and/or increased duration of treatment. Youth in foster care might also benefit from treatment approaches identical to evidence-based methods found for youth who are clinically referred. Independent of group affiliation, the youth in all three samples reported a broad specter of internalizing symptoms, indicating the need of treatments targeting different internalizing symptoms rather than treatment focusing specifically on a single subtype of internalizing symptoms. The transdiagnostic perspective proposes that there is more flexibility in contributing more universal treatment interventions and prevention strategies across specific disorders (61).

Apart from individual treatment of internalizing symptoms, interventions involving the close family relations of the youth have been proven helpful (73). Thus, interventions should be adapted to the needs of the individual youth as well as their families (54). An example of this is the Norwegian intervention program, “The Scaffolders” (our translation) (74), where clinicians systematically work with youth in foster care with complex problems and specifically aims to cooperate with the foster families as well as with the child-protective services. Although the project appears promising, it is not fully evaluated. Another family-based intervention, named “Alternatives for Families: a Cognitive–Behavioral Therapy,” involves the family of children exposed to abuse and other traumatic events to facilitate change in destructive relational patterns (75). However, interventions targeting children with trauma exposures vary in content, structure (individual or group-based), frequency, and duration and show varying effects (76). More research is needed to evaluate the effects of interventions toward at-risk youth in foster care and their families.

Nonetheless, the results generated from our study cannot inform us if youth in foster care have a qualitatively different symptom profile with a unique set of comorbidities compared to clinically referred youth. Future research is encouraged to investigate these matters to ensure that youth in foster care receive treatment tailored to their needs.

## Conclusions

The latent profile analyses on self-reported depressive symptoms and anxiety symptom subtypes across three youth samples (youth in foster care, youth in clinical treatment, and general population youth sample) show three symptom profiles. These profiles are characterized by differences in level and shape.

Prior exposure to maltreatment may be a leading factor in why youth in foster care are at a higher risk of belonging to the high symptom profile. Moreover, features within the foster care system itself may unintentionally contribute to a further increase in mental health problems in general, including internalizing problems—for instance, the consequences of a match or mismatch between the characteristics, needs, and capacities of the youth and the foster parents may affect a “failed” or “successful” placement (77, 78). The experience of foster parents, with follow-up and support from the child-protective services is identified to be essential in reducing the risk of placement disruption (78). Other systemic biases may therefore be placement instability and a high turnover among case workers, which can contribute to feelings of loneliness and a low or insecure sense of belonging. These may be altogether contributive factors for why youth in foster care are at a higher risk of belonging to the high symptom profile compared to their non-clinical peers.

It is critical that the services focus on the broad assessment of internalizing symptoms and early intervention, aiming to assess risk and protective factors in youth in foster care. Intervening early in the course of possible internalizing problems is crucial to prevent further disease progression. A transdiagnostic clinical staging model could be a promising framework for the management of these matters for youth in foster care. Overall, the findings indicate that current treatments may have to be tailored to the needs of youth in foster care.

## Data Availability Statement

The raw data supporting the conclusions of this article will be made available by the authors, without undue reservation.

## Ethics Statement

The studies involving human participants were reviewed and approved by The Norwegian Regional Ethics Committee West. Written informed consent to participate in this study was provided by the participants' legal guardian/next of kin.

## Author Contributions

SL, GW, and BH conceived the idea of this research project. SL initiated the methodological framework. YM, KB, GW, BH, ML, and SL contributed to the conceptualization and the design of the study. YM under the supervision of KB, conducted the statistical analyses and interpreted and presented the results in this manuscript. YM wrote the original main draft and was the main contributor to the present manuscript. GW, BH, KB, and SL contributed to the manuscript. ML critically commented on a draft. GW and BH took part in the data collections for the clinical and general population youth sample. ML and SL took part in the data collection for the foster care sample. All authors approved the submitted version of the manuscript.

## Conflict of Interest

GW reports receiving consulting fees and lecture fees from Medice. The remaining authors declare that the research was conducted in the absence of any commercial or financial relationships that could be construed as a potential conflict of interest.

## Publisher's Note

All claims expressed in this article are solely those of the authors and do not necessarily represent those of their affiliated organizations, or those of the publisher, the editors and the reviewers. Any product that may be evaluated in this article, or claim that may be made by its manufacturer, is not guaranteed or endorsed by the publisher.
